# A framework to improve parental participation in the hospital care of autistic children in the eThekwini district of KwaZulu-Natal

**DOI:** 10.4102/ajod.v15i0.1856

**Published:** 2026-05-14

**Authors:** Neil A. Williams, Dudu G. Sokhela, Thembelihle S. Ngxongo

**Affiliations:** 1 Department of Nursing, Faculty of Health Sciences, Durban University of Technology, Durban, South Africa; 2 Department of Health, KwaZulu-Natal College of Nursing, Durban, South Africa

**Keywords:** autism spectrum disorder, autism, child, framework, hospital, parent participation, nursing care

## Abstract

**Background:**

When children under 18 years old, diagnosed with autism spectrum disorder (ASD), autistic children are admitted to the hospital, they often have distressing experiences attributed mainly to overstimulation and communication issues. The quality of nursing care can be improved by effectively involving the child’s parents in their care. Currently, there is no framework to encourage parental participation in the hospital care of autistic children in South Africa.

**Objectives:**

The study explored professional nurses and parent perceptions on promoting effective parent participation in the hospital care of autistic children and to develop a framework for the improvement of parental participation in the hospital care of autistic children.

**Method:**

This study utilised the interpretative phenomenological analysis (IPA) research design, and data were collected using individual, virtual, semi-structured interviews. Employing the purposive sampling method, data saturation was reached with ten nurses and ten parents. Data were analysed using the IPA method of data analysis, and the framework was developed, utilising the Gray and Grove framework development process.

**Results:**

The study revealed 12 themes that were incorporated in a newly developed framework, under the following sub-concepts: Information sharing, proximity, attitude and garnering resources.

**Conclusion:**

This article presents a newly developed framework for the effective parent participation in the hospital care of autistic children.

**Contribution:**

The framework developed in this study can contribute to improving nursing practice for paediatric ward nurses and may improve the inpatient experience and health outcomes of autistic children and their parents.

## Introduction

When autistic children are admitted to hospital, they often have distressing experiences attributed mainly to overstimulation, communication issues and unfamiliar environments (Al Sharif 2025:1; Berglund 2017:89). This can lead to difficult behaviour, such as biting, scratching, crying and hurting themselves and others (Satopoh 2024:467). Lack of knowledge about autism by healthcare workers in hospitals may result in mismanagement of these children (Bernardo de Oliveira et al. 2025; Gautam 2020:17). Parental participation in hospital care can improve support for children with disabilities (Berglund 2017:89; Bono et al. 2022:377; Gautam 2020:17; Mimmo et al. 2019:1200).

Research shows that parental participation in autistic children’s hospital care is documented as essential to good health outcomes; however, this is still not being implemented (Fraatz & Durand 2021:1; Williams 2024; Williams, Sokhela & Ngxongo 2025). A lack of parent inclusion in the care of their hospitalised autistic child is a recognised problem globally, but there are only a few studies (Aderinto, Olatunji & Idowu 2023; Moosa-Tayob & Risenga 2022) that have been conducted in Africa and in South Africa. Morris, Greenblatt and Saini (2019:2374) state that there is a gap in knowledge concerning the nurse–parent relationship of autistic children in healthcare facilities and that it is possible that nurses themselves may have awareness of the obstacles and promoters of the nurse–parent relationship, and therefore their perceptions should be researched. Additionally, Shaibu et al. (2019:1616) suggest that because of perceived lack of parental participation, more research is needed to determine the most effective way that parents can be included in the care of their hospitalised autistic child. It is therefore evident that a gap in knowledge exists with regard to the perception of parents and nurses regarding parental participation in the hospital care of autistic children in South Africa.

### Research objective

The study explored professional nurse and parent perceptions on suggestions to encourage parental participation in the hospital care of an autistic child to ultimately develop a framework.

## Research methods and design

### Study design

The design for this research was an interpretative phenomenological analysis (IPA), underpinned by a relativist, interpretivist, socially constructed, inductive research approach (Williams 2024:207).

Interpretative phenomenological analysis explores the significance ascribed to the lived experience (Williams 2024:207), and admitting and autistic child to a hospital can be an extremely taxing experience for children, parents and the professional nurses. It is an experience that can only be described by individuals who have lived through it; hence, the IPA research design was used for this study Gray and Grove (2021), framework development process was utilised to develop a framework.

### Setting

The study was conducted in South Africa, in the KwaZulu-Natal (KZN) province and specifically in the eThekwini district. The eThekwini district was chosen because it is the most populated district in KZN and contains approximately a third of the entire population of KZN.

### Study population and sampling

To ensure triangulation of information, there were two populations for this study. The first population was parents of autistic children, whose child was admitted to the hospital in the above setting. The second population was professional nurses working in private paediatric wards in the above setting.

The purposive sampling method was utilised, and saturation was reached at ten parents and ten registered nurses. An advert (with inclusion criteria) was used to recruit participants; when a participant contacted the researcher, the researcher would assess if the participant met the inclusion criteria, and if the participant did meet the inclusion criteria, then informed consent was obtained from the participant, and an interview was scheduled. This was done until data saturation was reached. Purposive sampling was utilised because according to Frechette et al. (2020), it is a strategy that is commonly implemented in phenomenological studies because it permits the selection of participants who have in-depth knowledge of the phenomenon under study, and in this study, the researcher sorted the lived experiences of registered nurses and parents with regard to the hospitalisation of an autistic child.

### Data collection tools

The researcher developed one interview schedule for parent participants and a different interview schedule for professional nurse participants with six open-ended questions each. These interview schedules enabled detailed dialogue with participants to attain the study’s aim. The interview schedules have been checked for consistency and trustworthiness by two qualitative research experts, who served as supervisors for this research. These tools were pre-tested before the commencement of data collection on one nurse and a parent. Pre-testing data were not utilised in this study (Williams 2024:116).

### Data collection

Parent participants for the study were recruited through an advertisement shared on social media, namely Facebook^®^ and WhatsApp^®^ (Williams 2024:31), by the researcher and a non-profit autism organisation. Professional nurse participants were recruited via an advert sent to their employers and via social media (Williams 2024:32). Once the participant contacted the researcher, the researcher would assess if the participant met the inclusion criteria and then email the participants and information letter and consent document to sign and revert via email prior to the interview (Williams 2024:32). At the beginning of the scheduled interview, verbal consent was also obtained to conduct and record the interview. Data were collected from professional nurses and parent participants by the researcher between November 2021 and January 2022, which was during the COVID19 pandemic, and therefore online interviews were used to prevent accidental spread of this disease during the interviews. Data were collected using semi-structured interviews virtually on Microsoft Teams software (MS Teams^®^), on a date and time agreed upon with the participants. The interviews were recorded on MS Teams^®^with the permission of the participants (Williams 2024:35). Semi-structured interview guides facilitated discussions with participants to achieve the research objectives.

#### Data management and storage

The researcher has secured all data gathering tools, recordings and transcripts in a locked cabinet. The researcher will destroy these objects after keeping them under lock and key for 5 years. A code is used to identify all data gathering instruments, recordings and transcripts, of which only the researcher is aware of this code.

### Data analysis

The Peat, Rodriguez and Smith (2019:8) IPA data analysis method was utilised in this study (Williams 2024). This method has seven steps, which were implemented as follows: Step 1: The transcript was downloaded from MS Teams^®^ and uploaded to the Nvivo^®^ programme (version 12). Step 2: The researcher used Nvivo^®^ software to manually code the data bit by bit after intensely reading each transcript while listening to the recording multiple times (Peat et al. 2019; Williams 2024:37). Step 3: The researcher then used the software to look at the frequency of the codes and group the codes together, and themes started to emerge. Step 4: Groups of data or codes were analysed and determined interconnectivity (Peat et al. 2019). Step 5: The authors continued to the following transcript, ‘bracketing’, any previous themes, repeating steps 1–4 for successive transcripts (Peat et al. 2019). Step 6: When all transcript analysis has been concluded, collective themes are identified (Peat et al. 2019). Step 7: The authors subsequently analysed the collective themes to find the significance to the study (Peat et al. 2019). Conclusively, the authors examined current sources to validate the themes (Williams 2024:37). The data analysis was independently authenticated by two qualitative and nursing research experts.

### Trustworthiness

Trustworthiness of this study was achieved by adhering to the four criteria of trustworthiness as described by Lincon and Gruba (1985:301), namely credibility, transferability, dependability and conformability. Credibility was ensured by video recording the interviews, and verbatim notes were taken, preserving the participants’ real opinions (Williams 2024:37). To guarantee data saturation, probing questions were employed. Source triangulation was utilised to enhance credibility, drawing information from nurse participants, family participants and relevant literature. Additionally, the researcher maintained a presence in the field until data saturation was achieved, thereby facilitating a comprehensive understanding of the phenomenon (Williams 2024:37). There was a detailed explanation of the participants, methodology and research background, so that the readers can determine its transferability to other contexts. Dependability was ensured by asking the parents and nurses to evaluate all findings prior to submitting for publication, to ensure consistency over time, pretesting the data collection tools and transparency of findings. To ensure confirmability, objectivity and transparency, the data were made available to two nursing research experts to thoroughly examine and concur with the findings.

### Ethical considerations

Ethical clearance was obtained from the Durban University of Technology, (No. IREC 221/21), the relevant Department of Health (NHRD ref: KZ_202110_025), and selected hospital group (Approval Number: UNIV-2021-0058). The researcher disclosed the study’s nature, participants’ right to decline participation and withdraw at any time, and the risks and advantages. Agreeable participants read the information letter and signed the consent form and returned the form via email. Codes were used for confidentiality, and data were kept in a password-protected external hard-drive.

## Results

### Demographics

The demographics of the parent participants are represented in Table 1 and professional nurse participants in Table 2; both tables have been published previously in an article by the same authors: Williams et al. (2025:4).

**TABLE 1:** Parent participant demograhics (*N* = 10).

Participant	Age in years	Gender	Race or ethnicity	Marital status	Relationship to child	Age of child (years)	Employed outside home	Admitting diagnosis	How long ago was the admission
1	38	Female	African person	Married	Mother	4	No	Risperdal overdose	2 years
2	28	Female	African person	Single	Mother	16	Yes	Laceration of right hand	2 years
3	29	Female	Indian person	Married	Mother	6	No	MRI and audiology test	1 year
4	60	Female	Caucasian person	Married	Mother	18	No	Dehydration	3 years
5	40	Female	Indian person	Married	Mother	2	No	Chest infection	8 months
6	41	Female	African person	Divorced	Mother	18	Yes	Aggressive behaviour	4 years
7	50	Female	Indian person	Married	Mother	16	Yes	Incision and Drainage of Abscess	2 years
8	30	Female	Mixed race person	Married	Mother	7	No	Seizures	1 month
9	51	Female	Indian person	Married	Mother	17	Yes	Depression	2 years
10	45	Female	Mixed race person	Married	Mother	12	Yes	Dental procedure	2 years

*Source*: Williams, N.A., Sokhela, D.G. & Ngxongo, T.S., 2025, ‘Challenges hindering family involvement in the hospital nursing care of a child with autism’, *Health SA Gesondheid* 30, 9. https://doi.org/10.4102/hsag.v30i0.2731, p. 4

MRI, magnetic resonance imaging.

**TABLE 2:** Nurse participant demographics (*N* = 10).

Participant	Age (years)	Gender	Race or ethnicity	Marital status	Do you have children	Level of education	Years of service
1	53	Female	Indian person	Widowed	Yes	Diploma in general nursing	31
2	50	Female	Indian person	Married	Yes	Diploma in general nursing	11
3	50	Female	African person	Married	Yes	Diploma in general nursing	30
4	31	Male	Indian person	Single	No	Bachelor’s degree	10
5	33	Female	African person	Married	Yes	Diploma	13
6	35	Female	Mixed race person	Married	Yes	Diploma	15
7	40	Female	Caucasian person	Married	Yes	Diploma	10
8	30	Female	Indian person	Married	Yes	Diploma	10
9	36	Female	Mixed race person	Married	Yes	Diploma	4
10	42	Female	Indian person	Married	Yes	Bachelor’s degree	16

*Source*: Williams, N.A., Sokhela, D.G. & Ngxongo, T.S., 2025, ‘Challenges hindering family involvement in the hospital nursing care of a child with autism’, *Health SA Gesondheid* 30, 9. https://doi.org/10.4102/hsag.v30i0.2731, p. 4

### Themes

The authors will first present the themes for each participant group separately and then the themes that were common to both participants groups, will be discussed in more detail.

#### Subthemes for each participant group

Table 3 illustrates the subthemes for each participant group.

**TABLE 3:** Subthemes for each participant group.

Subthemes identified by nurse participants	Subthemes identified by parent participants
Need to improve own knowledge of Autism	Inadequate knowledge of autism by nurses
Communicating with parents ensures compliance	Poor communication by nurses
Listening to parents assisted in nursing care	Poor listening skills of nurses
Taking a detailed history of the child assists in planning nursing care	Nurses had inadequate knowledge of the specific child
Lack of an individualised nursing care plan	Using the child’s likes and dislikes to gain their cooperation
Parents are experts on their child and therefore must be involved in their child’s care	Parents desired more inclusion in their child’s hospital care
A good nurse–parent relationship is important	A weak nurse–parent relationship is bad for the child
Nursing children with ASD takes more time than nursing a neurotypical child	Some nurses have an impatient attitude
Parents should bring toys and entertainment for their own child	The hospital should provide toys and entertainment for the child with ASD
-	Harsh hospital environment for a child with ASD
-	Unmet nutritional needs of a child with ASD

*Source:* Williams, N.A., 2024, *An interpretative phenomenological analysis of family involvement in the hospital nursing care of children with autism spectrum disorder within eThekwini District*, pp. 132, Durban University of Technology, Durban

ASD, autism spectrum disorder.

#### Themes common to both participant groups

The authors have presented the subthemes and suggestions that were made by both the parent and professional nurse participants in Table 4. The subthemes and suggestions were grouped in themes that were guided by one of the theoretical frameworks of this study, namely: The theory of family care during critical illness by De Beer and Brysiewicz (2019). In this theory, the core concept is empowerment and is made up of four sub-concepts: ‘Information sharing, Proximity, Garnering Resources and Cultural and religious cooperation’ (Williams 2024:91). These concepts and this theory will be discussed in detail later in this article.

**TABLE 4:** Themes, subthemes and suggestions.

Theme	Subtheme or perception	Suggestion
1. Information sharing	1.1) Inadequate knowledge of autism	1.1) Nurses must be trained to nurse autistic children Williams (2024:132).
	1.2) Poor communication	1.2) Nurses must communicate often with parents Williams (2024:132).
	1.3) Poor listening skills	1.3) Nurses must listen to parents Williams (2024:132).
	1.4) Inadequate knowledge of the specific child	1.4) Nurses must take a detailed history of the autistic child Williams (2024:132).
	1.5) Lack of an individualised nursing care plan	1.5) Nurses must use the autistic child’s preferences to gain co-operation Williams (2024:132).
2. Proximity	2.1) Decreased parental participation	2.1) Nurses must improve parental participation
	2.2) A weak nurse–parent relationship	2.2) Nurses and parents must build a good nurse–parent relationship
3. Cultural and religious cooperation	3.1) Impatient attitude	3.1) The nurse must have a caring attitude and be patient when nursing an autistic child Williams (2024:132)
4. Garnering resources	4.1) Lack of therapeutic resources in hospital	4.1) Provide the autistic child with appropriate toys and entertainment during hospitalisation
	4.2) Harsh hospital environment	4.2) Accommodate the child’s sensory needs during hospitalisation
	4.3) Unmet nutritional needs.	4.3) Cater to the autistic child’s food preference

*Source:* Williams, N.A., 2024, *An interpretative phenomenological analysis of family involvement in the hospital nursing care of children with autism spectrum disorder within eThekwini District*, pp. 132, Durban University of Technology, Durban

#### Theme 1: Information sharing

According to the theory of family care during critical illness by De Beer and Brysiewicz (2019:22), information sharing is defined as the communication of truths about the patient and major illness among both nurses and parents, which increases everyone’s wisdom. Receiving information on a child’s status, care, progression and results broadens parent’s understanding, allowing them to best deal with a child’s illness (De Beer & Brysiewicz 2019:22). In the current study, the theme ‘information sharing’ incorporates the subthemes: Inadequate knowledge of autism, poor communication, poor listening skills, inadequate knowledge of the specific child and lack of an individualised nursing care plan.

**Subtheme 1.1: Inadequate knowledge of autism:** Parent participants thought that improved knowledge would improve awareness of the nurses, thereby translating into an improved nurse–parent relationship and better parental participation (Williams 2024:150). This was also supported by the nurse participants who perceived their understanding of autism to be minimal and wanted to be trained on nursing an autistic child (Williams 2024:151). The participants had the following suggestions:

‘The nurses must be educated on autism and have some sort of in-service training on autism. The nurses need more education on autism,’ (Parent participant 2)

‘Nurses need to have in-service training or readable literature informing them of this diagnosis. If we are equipped with that knowledge, the task becomes easier to deal with. With knowledgeable nurses, parents will be at ease when we take care of their kids and better informed. The severity of the cases differs, so knowledge is key,’ (Nurse participant 8)

**Subtheme 1.2: Poor communication:** Some parents reported bad occurrences with ineffective communication by hospital staff (Williams 2024:154). Parent participants believed their expert knowledge of their child was being ignored, leading to a difficult hospitalisation experience (Williams 2024:154). Some of the nurse participants agreed that if the nurse effectively communicated with the family, this would promote effective family involvement:

‘Nurses must communicate with the parent more so they can understand the child,’ (Parent participant 1)

‘I think the most important thing for us as nurses is to communicate with the parents; communicate with the parents and discuss information with the parents,’ (Nurse participant 2)

**Subtheme 1.3: Poor listening skills:** ‘Nurses must listen to parents more’. Parents perceive nurses as experts in their children’s health, and when they are heard, they feel respected and cared for. This promotes effective parental participation; however, when nurses don’t listen, it is seen as disrespectful and arrogant. Conversely, when nurses listen, it fosters teamwork and promotes parental participation in the hospital care of autistic children. Nurse participants found listening to parents as a coping strategy to overcome their inadequate knowledge of Autism, enhancing the child, parent and nurse experience. They saw the parents as a knowledgeable partner, leveraging their expert knowledge for better nursing care:

‘The nurses must listen to what the parents are saying and not be offended because the parents know their child better than the nurses. They must not assume that the child is naughty but ask the parent what to do,’ (Parent participant 1)

‘Firstly, they must listen to the parents they will tell you the dos and don’ts. Let the parents’ guide you. “Listen to the parents. The parents can teach us what works at home with the child. The parents can give us information like what the child likes and dislikes, so we can use the likes in the nursing care of the child. The parents can tell us if we can play with the child and some of the child’s behaviour like if he or she screams, bites, injures themselves and let the staff know if they do,”’ (Nurse participant 9)

**Subtheme 1.4: Inadequate knowledge of the specific child:** All participants agreed nurses needed to obtain a thorough medical history from the parents so that they would understand how to manage them correctly. From the following quotes, it is apparent that parent participants believe that taking a detailed history of their autistic child promotes effective parental participation and quality nursing care. They believe that detailed history allows nurses to plan care appropriately, meet their child’s special needs and ensure a pleasant hospital experience. This approach is seen as a caring attitude and willingness to learn. Nurse participants viewed detailed history taking as an empowering exercise to gather information about the autistic child, gain trust and involve parents in their care. They acknowledged the distinctiveness of every child and the significance in understanding their unique circumstances for effective nursing care:

‘I think firstly the nurses should have found out how I take care of my son at home, how I calm him down at home, what triggers aggressive behaviour in him, they should have found out about what he likes to eat. Get a detailed history from the parent,’ (Parent participant 6)

‘Taking a good history from the parents is very important, especially about who the child responds to et cetera, ask the parents what triggers the child and what calms him down,’ (Nurse participant 1)

**Subtheme 1.5: Lack of an individualised nursing care plan:** Parent participants suggest that nurses should understand their children’s likes and dislikes, creating a suitable physical and psychological environment for their autistic children. This individualised care demonstrates a caring attitude towards the children and their parents. Nurse participants agree that understanding a child’s likes and dislikes is crucial for effective parental participation. This knowledge helps nurses provide better care, maintain parent and child happiness and deliver good nursing care. They acknowledge that each autistic child is unique and needs to be understood to plan effective nursing care and involve parents:

‘Music calms him down, they did not have any music to calm him down. I think firstly the nurses should have found out how I take care of my son at home, how I calm him down at home, what triggers aggressive behaviour in him, they should have found out about what he likes to eat. Try and keep to the child’s routine.’ (Parent participant 6)

‘The parents can teach us what works at home with the child. The parents can give us information like the child likes and dislikes, so we can use the likes in the nursing care of the child. The parents can tell us if we can play with the child and some of the child’s behaviour like if he or she screams, bites, injures themselves and let the staff know if they do,’ (Nurse participant 9)

#### Theme 2: Proximity

De Beer and Brysiewicz (2019:22) define ‘proximity’ as the nurse’s readiness to assist the parent’s access to their child (Williams 2024:190). The nurse should facilitate parent’s physical and emotional closeness to the child, permitting them to monitor the situation, follow the treatment given to the child and establish confidence and trust in the care received by the patient (De Beer & Brysiewicz 2019:22). When parent’s entry to their admitted child is limited, unpleasant feelings like dread, worry and powerlessness are exacerbated (De Beer & Brysiewicz 2019:22). Permitting parents to be near their child lessens these bad feelings as they may give assistance without sacrificing closeness (De Beer & Brysiewicz 2019:22). This sub-concept of proximity incorporates this study’s subthemes, decreased parental participation and a weak nurse–parent relationship.

**Subtheme 2.1: Decreased parental participation:** Parent participants express a desire for increased participation in their child’s care, including consultation on healthcare decisions. Parents require acknowledgement as expert partners (Williams 2024:144), and their concerns should be considered by nurses. The nurse participants also made this suggestion and emphasised the significance of parental participation in the hospital care of autistic children, as they believe parents are experts and can assist in the child’s care (Williams 2024:144):

‘I believe getting the parents involved in caring for their child, will help develop a good relationship between the nurse and the parents,’ (Parent participant 7)

‘I think it is important to involve parents every step of the way. Involve them in the nursing routine. Involve the parents in the patient’s care. Get the parents involved to calm the child down if they are upset,’ (Nurse participant 1)

**Subtheme 2.2: A weak nurse–parent relationship:** The parent and nurse participants both agreed that developing a courteous connection between the parents and nurses is beneficial for all concerned and will also improve parental involvement in the hospital care of autistic children:

‘The nurses need to build a relationship with the child and the parents,’ (Parent participant 3)

‘I also think that if we speak to the nurse well and develop a good relationship with them, things will go well. Develop a good relationship with the parents. Parents must speak to the nurses and work together with them,’ (Parent participant 7)

‘Families must work together with the nurses, for example parents tell us what’s the child’s likes and dislikes because the parent knows the child best. Get information from the parent. The parents help give the medication, so that before discharge the parents must understand the medication and be totally involved in the care and management of the child. The parents always stay with the child,’ (Nurse participant 6)

#### Theme 3: Cultural and religious cooperation

When nurses demonstrate awareness, respect, understanding and acceptance of parents’ attitudes, values, beliefs and practices throughout their children’s hospital admission, this is referred to as cultural and religious cooperation (De Beer & Brysiewicz 2019a:23). In this study, the subtheme of impatient attitude is incorporated under this theme.

**Subtheme 3.1: Impatient attitude:** Many parents had experienced discrimination from nursing staff because of their child’s autism spectrum disorder (ASD) and perceived nurses as impatient, cold, unfriendly and aggressive. They suggested that a caring, friendly, understanding and patient attitude can create a more conducive environment, build better nurse–patient/nurse–parent relationships and promote parent participation for hospitalised autistic children. The professional nurses also agreed with this suggestion, saying:

‘I expected the nurses to be more patient and to help the parents to care for the child. The nurses need to be calm and friendly with the parents and children. They need to build a relationship with the child and the parents,’ (Parent participant 3)

‘I think the most important thing is for the nurses to have patience with this child. The nurse needs to understand the child; the nurse must have patience with the child. The nurse must be patient with the parents and the child. Nurses must also understand that these children’s behaviour is different and difficult at times,’ (Nurse participant 3)

#### Theme 4: Garnering resources

De Beer and Brysiewicz (2019:22) define ‘Garnering resources’ as parents and an autistic child receiving the resources they require. These resources are required so they can meet their basic physiological and psychological needs such as food, sleep and hope (De Beer & Brysiewicz 2019:22; Williams 2024:190). Parents and autistic children require non-material resources such as people to share their pain with (Williams 2024:190). Parents and autistic children also require a comfortable waiting area, where they can get enough rest and sleep, if feasible (Williams 2024:190). Parents frequently feel compelled to remain close to the child; therefore, it is critical to have easy access to water and food in the waiting room (De Beer & Brysiewicz 2019:22). This sub-concept incorporates subthemes: Lack of therapeutic resources in the hospital, harsh hospital environment and unmet nutritional needs

**Subtheme 4.1: Lack of therapeutic resources in the hospital:** Autistic children have a short concentration span and become irritable in an unfamiliar environment. Parent participants believe that children need toys or entertainment in hospitals to promote effective family involvement. Parent participants explained how they had created a soothing home environment by using toys and entertainment, which reduced parental stress.

However, many parent participants verbalised a lack of toys and entertainment (distractions) in the hospital, which caused stress for the child and parents. Older participants brought entertainment like iPads with music to keep the child entertained. Therefore, having toys or entertainment is crucial for a child’s comfort and well-being. Nurse participants agree that toys and entertainment are crucial for children with ASD when admitted to the hospital. They recognise the value of play in treating children and see them as positive distractions. Nurse participants believe that it is the duty of parents and management to provide these toys (Williams 2024:169), addressing the child’s sensory challenges in a new environment. Following are some verbatim quotes from some participants:

‘The paediatric ward should have a playroom with sensory toys to use in the playroom to calm the child down and be a form of distraction,’ (Parent participant 5)

‘The hospital was not child friendly at all, there were no toys or any distractions to entertain my daughter. They should offer toys or other distraction for the child,’ (Parent participant 8)

‘I remember that in one case the parents used the music and videos on their cell phone to help calm and entertain the child, with another child the parents brought the child’s bunny and then the doctor and nurses examined the bunny first and then examined the child. I remember it was a very long consult but it worked very well,’ (Nurse participant 2)

‘Dealing with ASD kids, we need to create a playful and safe environment. We need to be at the level of the child. We need to interact with the parents. The nurse must get the child to trust you and must not see you as a treat, you can play with the child,’ (Nurse participant 6)

**Subtheme 4.2: Harsh hospital environment:** All parent participants suggested that the autistic child be nursed in a private room to cater to the sensory needs of the child. It was noted that those parents and children who were given private rooms had a good lived experience and vice versa:

‘We were put in a private room, which was very good because I kept the curtains closed and lights off because the light affects my son, who is sensitive to bright lights. The child must be admitted in a private room because of the sensory issues of children with ASD, the curtains, room lights, et cetera, are important,’ (Parent participant 5)

**Subtheme 4.3: Unmet nutritional needs:** Autistic children are known to have restricted diets because of sensory problems, and many parents recounted the experience of their child not eating the food in the hospital, which the parent participants perceived as the uncaring hospital not meeting the child’s basic need (Williams 2024:178). Parent identified this as a major concern for them. Parents also observed this to be ignorance of the special sensory and dietary requirements of their child (Williams 2024:178):

‘He did not eat at all in the hospital and did not like any of the food they offered so he did not eat all day. They need to ask what food the child eats, so they can make that food for the child,’ (Parent participant 3)

‘The menu did not cater for my son and the food he likes to eat, so we bought our own food for him. They should ask what does the child like to eat. They should accommodate the food preference of the child,’ (Parent participant 4)

## Discussion

The findings of this study highlighted the lived experiences of parents and professional nurses, the majority of which were negative experiences. The negative perceptions of parents and nurses were grouped in four themes, namely: information sharing, proximity, garnering resources and cultural and religious cooperation (De Beer & Brysiewicz 2019:22; Williams 2024:91). As highlighted in the findings, both groups of participants also made suggestions on how to improve parental participation in the hospital care of an autistic child. These twelve suggestions will be discussed as follows.

### Theme 1: Information sharing

In a recent qualitative systematic review, Barratt et al. (2024:936) corroborated the findings of this study, in that nurses expressed a need for more training after identifying gaps in their education and expertise on the care of autistic children. Barratt et al. (2024:940) also found that nurses who were better educated and trained were able to deal with children and their parents more successfully because they could show off their knowledge and abilities, giving parents in particular a sense of security and support. Mahoney et al. (2021:8) agree with the finding of this study that communication is an important component in promoting effective parental participation. According to Mahoney et al. (2021:8), parents perceived good communication as a facilitator of good care and the converse being true as well (Williams 2024:154). Effective continuous communication between nurses and parents helps to build a trusting relationship because parents appreciated nurses communicating with them and their child and listening to them (Marino et al. 2023:1). Walsh et al. (2020:422) also found that ‘Not listening to parents’ is a barrier to parental participation in the hospital care of their autistic child, and Nicholas et al. (2020:95) corroborated this finding by stating that parents’ opinions and expertise should be valued and heard, as this leads to the provision of effective, individualised care for autistic children and their parents. Harvey et al. (2023:912) agree with the finding of this study that nurses should gather a detailed history from the parent of an autistic child in order to better understand the child and manage the child and increase parental participation.

The participants of this study highlighted the need for using the autistic child’s preferences to gain their co-operation, and this was also echoed by Owen, Gary and Schnetter (2020:35) who stated that good nursing care involves putting autism knowledge into practice and listening to the parents of the autistic child about the child’s preferences. Magalhães et al. (2020:558) also agrees by noting that the child’s likes and dislikes can be used to develop different strategies in the management of the autistic child, with the purpose of promoting successful nursing care, such as the musical intervention and the use of toy resources, which are a very important nursing task.

### Theme 2: Proximity

All children have the right to family or parental care under Section 28 (1)(b) of the South African Constitution (Williams 2024:165). This right also applies to hospitalised children who need support from parents and family while they are in the hospital (Mahery 2021:1). Increased parental participation in children’s health care is anticipated to increase the quality of care and safety for children, because parents have valuable knowledge about their child and are important helpers in implementing their children’s health care (André et al. 2025). The sentiments of the above authors are echoed in the perceptions of the participants of this study. As in this study, Barratt et al. (2024:940) found that creating respectful and supportive relationships between hospitalised children, their parents and nurses is a key component of parental participation (Roquette Viana et al. 2021) and also noted that parents are empowered to get involved in their child’s care when there is a positive nurse–patient relationship and knowledge sharing is in place.

### Theme 3: Cultural and religious cooperation

Nurses must have a caring attitude and patience, which was the finding of this study and was corroborated by Eben (2024:103) who found that the attitude of nurses towards autistic children affects the care they give to the children and their parents. Coşkun et al. (2025:137) also noted that the nurse’s attitude towards parent’s participation is vitally important in ensuring its success.

### Theme 4: Garnering resources

#### The child needs toys or some entertainment in the hospital

According to Litwin and Sellen (2023), although there is substantial individual variance in preferences and aversions, the majority of children and teenagers with ASD exhibit sensory sensitivities, and many healthcare settings are not suited to adequately meet their requirements.

In an integrative review of recent literature conducted by Magalhães et al. (2020:557), the authors reported that nurses found the following strategies help improve the nursing care of the child with ASD: (1) strategies that enable the insertion of playful experiences as a way to promote care; (2) musical intervention as a technique of caring for children with ASD, which can provide a moment of creative interaction, stimulate communication and change the behaviour of these children; (3) the use of ludic resources (toys), which are used by nursing professionals to encourage the child’s sense of autonomy, communication and behaviour change through creative interaction. Drake et al. (2012:215) conducted research in which they deployed a coping kit for paediatric inpatients and examined the cost-effectiveness and nurse assessment of the kit’s usefulness, finding that it was a very successful tool embraced by autistic children, parents and nurses.

#### Safety of autistic child and other children

Autistic children have a higher rate of injury and a higher death as a result of injuries; therefore, nurses who care for children with ASD must be ready to discuss safety with the parent and provide a safe environment for the child (Celia et al. 2020:222). The primary caregiver parent is an expert on their child and a vital source of knowledge, and all medical personnel should recognise this, especially when it comes to safety (Celia et al. 2020:228). McIntosh and Thomas (2020:2) explain that autistic children can be exposed to unintentional injuries, for example: Needles, tablets, bodily injury, food and too much stimulation during hospitalisation.

McIntosh and Thomas (2020:2) warn that it is important to take safety precautions while changing the surroundings, which should include securing sharps containers to prevent children from sticking their hands inside them, securing sharp items in a cupboard (Williams 2024:174). Minimal furniture and removal of dangerous medical equipment from the room may prevent injuries to the child (Williams 2024:174). To prevent unintentional drug intake, all cabinets must be secured, as people with ASD may try to open drawers and cabinets to start sorting the contents (McIntosh & Thomas 2020:2).

#### Food requirements for a child with autism spectrum disorder

According to Mendive Dubourdieu and Guerendiain (2022:1), it is a well-known and researched fact that many children with ASD follow a gluten- and casein-free (GCF) diet or have a limited diet because of sensory issues. Nurses should therefore discuss dietary requirements with parents on admission of the child to the hospital (Williams 2024:179).

### Motivation for the development of a framework

This research showed that some parents had negative encounters, while others had positive encounters, revealing a lack of uniformity in parental participation in hospital care of autistic children and therefore the need for standardisation, which can be achieved through the formulation and implementation of a framework (Williams 2024:184). This research also identified the perceived challenges to the parental participation in the hospital care of autistic children, which was the first step in effectively overcoming these barriers; the next step would be the development of a framework to overcome these challenges; and the final step would be implementing and evaluating this framework (Williams 2024:184). The suggestions reported by the participants were vital in the promotion of effective parental participation; nonetheless these needed to be grounded in established and verified theories; therefore, a framework was developed to incorporate this empirical data with established theory to construct a new framework that could promote effective parental participation in hospital care of autistic children.

### Framework development process

The framework for the improvement of parental participation in the hospital care of an autistic child (Williams 2024:187) was created by combining research’s data from this study and Neuman’s systems model, Bowen’s family systems theory and the theory of family care during critical illness (Williams 2024:203). The newly developed framework was authenticated by the ‘Delphi’ method of validation, utilising a panel of experts. The expert panel is composed of One medical doctor with knowledge of autistic children, two professional nurses with a post-basic qualification in child nursing science, two professional nurses working in hospital nursing autistic children and two members of autism support group (Williams 2024:198). A consensus Delphi was reached after two rounds.

The researcher followed the process of framework development as described by Gray and Grove (2021:182), namely: Defining relevant concepts, developing relational statements and constructing a conceptual map.

#### Step 1: Defining relevant concepts

According to Gray and Grove (2021:182), the first step in framework development is defining the relevant concepts. According to these writers, concepts are selected for the framework based on their applicability to the issue statement, literature and phenomenon of interest. Every concept in a framework needs to have a conceptual definition and be able to be found in previously published theoretical works (Williams 2024:188).

The following concepts are included in the framework:

- **Autistic children:** The autistic child in this research is defined as a person under the age of 18 years old who has been diagnosed with having ASD (Hughes et al. 2023:971; Lansdown & Vaghri 2022:407).
- **Nurse:** The NSM views nursing as a unique profession that is concerned about the patient as a whole and all variables in the client’s environment that may affect them, including the nurses’ perception (Lawson 2021:234).
- **Parent:** Parents are usually the biological mother and father of the autistic child admitted to the hospital (Williams 2024:189).

#### Suggestions to promote effective parental participation

Eleven suggestions emerged from the empirical findings from this study; to improve parental participation and effective family involvement, these suggestions were amalgamated into the relevant sub-concepts of empowerment in the process of developing a framework.

#### The core concept: Empowerment

De Beer and Brysiewicz (2019:22) explain that empowerment consists of the sub-concepts of Information sharing, proximity, garnering resources and cultural and religious cooperation (Williams 2024:190). These four sub-concepts were discussed in detail, in the findings section and therefore will not be discussed again here.

#### Sub-concept: Garnering resources

Parents frequently feel compelled to remain close to the child; therefore, it is critical to have easy access to water and food in the waiting room (De Beer & Brysiewicz 2019:22). This sub-concept incorporates subthemes: The hospitalised child needs entertainment, safety, accommodation of their sensory needs, suitable food, and a quiet environment (Williams 2024:190).

#### Step 2: Developing relational statements

Gray and Grove (2021:183) described how the next step in the framework development process is to connect all the study ideas through relational statements. To combine research findings, the researcher must establish evidence that there are connections between some or all of the concepts (Williams 2024:191). Relational statements connect concepts. The relational statements for this study have been described in the subheadings that follow (Williams 2024:191).

**Relationship between the nurse and the autistic child:** Neuman’s system model (NSM) shows that the nurse has a direct impact on the autistic child as the child relies on the nurse to regulate stressors in the hospital setting and thereby aid the child in reaching a state of wellness. The relationship between the nurse and the child is clearly illustrated by this framework (Williams 2024:191).

**The relationship between the parent and child with ASD:** With the Bowens Family Systems Theory (BFST), the parent has a direct impact on the autistic child, as well as the child’s health, and can be represented as follows (Williams 2024:192):

**The relationship between the nurse, parent and autistic child:** Pfeiffer and In-Albon (2022:186) point out that a ‘triangle’ – three people – is the smallest component of a family system in BFST (Williams 2024:193). A three-person relationship is more stable as a two-person relationship is too little and stress may develop quickly. The friction shifts between the three persons when a third person enters the relationship, in order to maintain the stability of all relationships (Williams 2024:193). If tension between the two closest members, referred to as the ‘insiders’, rises, they can choose to get closer to the third member, referred to as the ‘outsider’ (Williams 2024:193). Any conflict or tension that develops between any two people in a triangle tends to change the dynamics of that triangle (Pfeiffer & In-Albon 2022:186). Because everyone involved – the child, the nurse and the parent – perceives hospitalisation as a period of worry and stress, the child, nurse and parent would create Bowen’s notion of the triangle relationship in the present investigation. In the event of hospitalisation, the parent may help alleviate the stress on the nurse and vice versa (Williams 2024:193).

#### Step 3: Constructing a conceptual map

A conceptual map, according to Gray and Grove (2021:184), is a graphical depiction of a research framework. This conceptual map, also known as the relational statements, shows the connection between all the key ideas in the research in a visual manner (Williams 2024:193).

**FIGURE 1:**
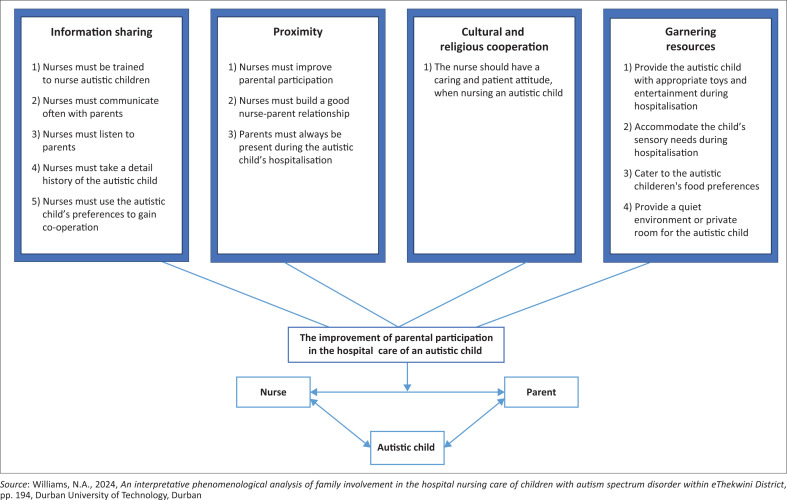
The framework for the improvement of parental participation in the hospital care of an autistic child.

Figure 1 is the conceptual map, ‘The framework for the improvement of parental participation in the hospital care of an autistic child’.

### Limitations

The framework’s primary drawback is that it is based on data obtained from a sample that is restricted to a single province in South Africa; thus its use may be constrained in explaining parental involvement in the hospital care of an autistic child in other provinces of South Africa, both domestically and abroad (Williams 2024:203).

## Conclusion

The study results show that the four empowerment factors – information sharing, proximity, attitude and resource acquisition – can be used to empower nurses and parents, which in turn can help to strengthen parental participation (Williams 2024:205). The framework created in this research has the potential to advance nursing care for nurses in the eThekwini area of KZN, who work in children’s wards, as well as perhaps improve the in-patient experience of autistic children and their parents (Williams 2024:205). If implemented correctly, it can help ensure that parents are meaningfully involved in their children’s treatment, which may result in better health outcomes for autistic children and more positive work experience for nurses in this area (Williams 2024:205).
